# Nonconvective Forces: A Critical and Often Ignored Component in the Echocardiographic Assessment of Transvalvular Pressure Gradients

**DOI:** 10.1155/2012/383217

**Published:** 2011-10-07

**Authors:** Michael S. Firstenberg, Erik E. Abel, Thomas J. Papadimos, Ravi S. Tripathi

**Affiliations:** ^1^Division of Cardiac Surgery, The Ohio State University Medical Center, Columbus, OH 43210, USA; ^2^Department of Anesthesiology, The Ohio State University Medical Center, Columbus, OH 43210, USA

## Abstract

Echocardiography is routinely used to assess ventricular and valvular function, particularly in patients with known or suspected cardiac disease and who have evidence of hemodynamic compromise. A cornerstone to the use of echocardiographic imaging is not only the qualitative assessment, but also the quantitative Doppler-derived velocity characteristics of intracardiac blood flow. While simplified equations, such as the modified Bernoulli equation, are used to estimate intracardiac pressure gradients based upon Doppler velocity data, these modified equations are based upon assumptions of the varying contributions of the different forces that contribute to blood flow. Unfortunately, the assumptions can result in significant miscalculations in determining a gradient if not completely understood or they are misapplied. We briefly summarize the principles of fluid dynamics that are used clinically with some of the inherent limitations of routine broad application of the simplified Bernoulli equation.

## 1. Introduction

Echocardiography has become an invaluable tool for the qualitative and quantitative assessment of cardiac function. The ability to evaluate ventricular function and valve pathology in real time, its portability, lack of ionizing radiation, and relatively low cost are all factors that have contributed to echocardiography becoming more common in the physiologic and hemodynamic assessment of sick patients. While 2-dimensional and, more recently, 3-dimensional imaging of cardiac structures are part of a routine qualitative assessment; both continuous and pulsed wave Doppler are often used for quantitative assessment of intracardiac flows depending on the magnitude of flow velocity and the need for spatial resolution [1]. While Doppler waveforms are routinely used to determine the magnitude of normal, regurgitant, and stenotic (restrictive) intracardiac flow, the limitations of the assumptions of the equations routinely used are rarely considered [2]. 

## 2. Theoretical Basis for the Assessment of Intracardiac Pressure Gradients

A primary application of the pulsed Doppler waveforms has been the estimation of pressure gradients, typically across both native and prosthetic valves [3]. The theoretical basis of this stems from the Navier-Stokes equations for incompressible fluid that describes three-dimensional flow [4]. The Navier-Stokes equations can then be rewritten and simplified to describe two-dimensional flow across a streamline; this is known as Euler's equation (1) and relates the instantaneous local pressure (∂*p*) and velocity (∂*v*) relationships as a function of distance (∂*s*) and time (∂*t*)

(1) $$\frac{\partial p}{\partial s}=-\rho \left[\frac{\partial v}{\partial t}+v\frac{\partial v}{\partial s}\right].$$

Integrating Euler's equation between 2 points along a pathway (such as within the heart between a point in the left atrium (*S*_LA_) and ventricle (*S*_LV_)) results in the unsteady Bernoulli equation

(2) $$\Delta p=\frac{1}{2}\rho \left(v_{2}^{2}\left[t\right]-v_{1}^{2}\left[t\right]\right)+\rho \overset{S_{\text{LV}}}{\underset{S_{\text{LA}}}{\int }}\frac{\partial v\left[s,t\right]}{\partial t}ds+G.$$

Similarly, the unsteady Bernoulli equation (also known simply as “the Bernoulli equation”) can be rewritten as

(3) $$\Delta p\left(t\right)=\frac{1}{2}\rho \Delta \left(v_{2}^{2}-v_{1}^{2}\right)+M\frac{dv}{dt}+R\left(v\right)+G,$$

in which Δ*p*(*t*) is the drop in pressure as a function of time (*t*) between the two points of interest, *ρ* is blood density (1.05 g/cm^3^), *v* is the velocity of blood between the two points of interest (*v*_1_ and *v*_2_), and *M* is the inertance term of blood flow. *R* is a resistive term reflecting the effects of viscosity along the path. *G* is the term that describes the effects of gravity and, in the context of intracardiac blood flow, is considered negligible and is typically ignored [5]. Similarly, since these applications often describe blood flow originating in either the right or left atrium for the assessment of transtricuspid or transmitral applications, the initial velocity within the left atriums have also been shown to be negligible and can be ignored [6]. This assumption of an initial minimal upstream velocity is a recurrent source of error in the assessment of intracardiac blood flow, regardless of the equations used. 

The complete form of the unsteady Bernoulli equation (3) consists of 4 terms [6] that describe the contributions of different forces that determine a pressure gradient:

1. a convective term (Δ*p*_conv_ = (1/2)*ρ*Δ(*v*_2_^2^ − *v*_1_^2^)) accounts for the fall in pressure and the simultaneous rise in the kinetic energy as fluid (i.e., blood) increases velocity across an orifice (i.e., a valve);
2. an inertial or nonconvective term (Δ*p*_int _ = *M*(*dv*/*dt*)) describes the pressure change that is required to accelerate a mass of blood across the valve;
3. a gravitational (*G*) term that describes the effects of gravitational forces on the mass;
4. a viscous term (Δ*p*_visc_ = *R*(*v*)) that describes the loss of energy from the viscous interactions between the fluid/blood along the walls.

## 3. Resistive or Viscous Forces

The contribution of resistive or viscous forces is based upon the Poiseuille equation  (4)

(4) $$\Delta p_{\text{visc}}=\frac{4\mu V_{\max\;}L}{r^{2}}.$$

The viscous resistance to flow (Δ*p*_visc_) is a function of the viscosity of blood (*μ*), the peak velocity (*V*_max_), and the length of the column (*L*), divided by the radius squared (*r* ^2^). For cardiac applications, and assuming steady-state laminar flow, for the range of human cardiac output (2–6 liters/minute), over the distance measured (typically only several centimeters within the heart), and for the range of valvular or tubular diameters (also typically only several centimeters), the contribution of the resistance term ranges from 0.006 mmHg (for a radius of 0.7 cm and a cardiac output of 2 liters/min) to 0.14 mmHg (for a diameter of 1.0 cm and a cardiac output of 6 liters/min) [5]. Hence, as mentioned, in the context of intracardiac flow and pressures, viscous forces are considered negligible and are typically ignored.

## 4. Inertance (Nonconvective) Forces

The inertance term, *M*, is a function of the energy required to accelerate a mass of blood (*dv*/*dt*), and it can be described by rewriting the unsteady Bernoulli equation

(5) $$M\;\approx \frac{{\Delta P}_{\text{act}}\;-{\Delta P}_{\text{conv}}}{dv/dt}.$$

*M* can be approximated as the difference between the actual pressure gradient (Δ*P*_act_) and the convective component of the pressure gradient (Δ*P*_conv_ = (1/2)*ρ*Δ[*v* ^2^]) as a function of the changes in velocity over time (again assuming negligible viscous and gravitational forces). For routine applications, *M* is also typically ignored because it requires being able to derive the change in velocity (acceleration) over a distance, a task that is very difficult to accurately accomplish when measuring intracardiac blood flow. This spatial acceleration is not available by conventional 2D Doppler velocity data (which only provides the velocity characteristics of blood at a point/region of interest). Color M-mode Doppler, unlike conventional Doppler imaging that provides a velocity at a specific point within the heart, provides encoded velocities over an entire scanline with the colors displayed directly correlating to that specific velocity on the scanline. The scanline provides the distances while real-time recording adds the component of time. Recording these scan-line velocity characteristics over time allows for determining the nonconvective or inertial forces [7]. While sophisticated analysis of color M-mode imaging has demonstrated the ability to determine the spatial acceleration of blood and the inertial component, these tools are not easily available and therefore not part of routine clinical applications. Furthermore, acquisition of these images requires the scan-line to be directly oriented in a 90 degree angle to the direction of flow to prevent underestimation of velocities by off-angle measurements. While in theory Doppler scan-line orientation can have a significant impact on underestimating true velocities, and hence true pressure gradients, the magnitude and significance of off-angle measurements are unclear [8]. Furthermore, in routine clinical applications, either with transthoracic or transesophageal imaging, the ability to accurately orient the Doppler scanline in a patient can be technically challenging. Much like gravitational and viscous forces are often considered negligible as are inertance forces, in part because of the difficulty in measuring them accurately; however, inertance forces have been shown to be physiologically complex, incompletely understood, and considerably variables in ways that can lead to a substantial underestimation of the overall pressure gradient which needs to be considered [9]. 

For routine clinical applications, the estimation of a pressure gradient within the heart typically only considers the easy-to-measure convective term. It is this convective term that is commonly referred to as the “modified” Bernoulli equation that is the complete Bernoulli equation (3) minus the inertial, gravitational, and viscous/resistance terms. When converted into appropriate scientific units, it becomes familiar: Δ*p* = 4*v* ^2^ [3], in which *v* is the Doppler-derived velocity in m/sec and Δ*p* is the estimated pressure gradient in mmHg. Again, an additional assumption is that the initial velocities, for example, in the left atrium, are minimal, and hence, only the final velocity is considered—a concept that is not necessarily valid.

For nonrestrictive orifices, such as a normal valves and “larger” conduits, these assumptions do not apply. Because of the relatively large amount of blood that must pass through a nonrestrictive mitral valve with each cardiac cycle, the inertial term is presumed to play a significant role in describing the overall transmitral pressure gradient [10]. Although previous investigators have demonstrated the importance of the inertial component of the Bernoulli equation when applied to transmitral flow, it is a term that is commonly ignored both clinically and in research [11, 12].

## 5. Clinical Data

Animal and human data regarding the absolute or relative contributions of transvalvular inertance and nonconvective forces are limited, with most work having been performed in the context of transmitral valvular pressure gradients (transaortic gradients and velocities are much higher and the inertial components contribute relatively less and probably have less clinical significance). Human* in situ *experiments, with high-fidelity pressure transducers placed across the mitral valve, in which actual pressure gradients are compared with the convective components (as determined using the modified Bernoulli equation from echo Doppler velocities) under a wide-spectrum of physiologic conditions, are used to estimate the inertance components [13]. In these human experiments, the actual catheter derived transmitral pressure gradients ranging from 1.04 to 14.24 mmHg. However, using simultaneously derived Doppler velocity, the inertance component (*M*(*dv*/*dt*)) ranged from 0.6 to 12.9 mmHg. A previously validated numerical model of the cardiovascular system [14] was then used to predict those physiologic and echocardiographic determinants of *M* (not to be confused with the complete inertial component, *M*(*dv*/*dt*)) [11]. The results of mathematical modeling demonstrated, using a multivariate analysis, that the strongest predictors of transmitral *M* was (1) maximum left atrial volume (an index of the “mass” of blood that needs to be accelerated) and (2) the ratio of pulmonary venous *S*/*D* wave velocities (an index of the initial kinetic/potential energy of blood within the left atrium that needs to be moved across the mitral valve). Overall, the inertial energy, on average, consisted of 74% of the actual pressure gradient as predicted using only the convective term [13].

In a similar set of human experiments, in 8 patients undergoing cardiac surgery, 56 cardiac cycles from 16 hemodynamic stages were studied. Actual pressure gradients were recorded with high-fidelity multisensor pressure transducers (Millar, Houston, Tex, USA) across a normal mitral valve. These actual gradients were correlated with noninvasive echocardiograph color M-mode images. This study demonstrated, for a large range of physiologic conditions, that the Δ*p*_conv_ consistently underestimated Δ*p*_act_ (*r* = 0.72, *P* < 0.05), and, in fact, Δ*p*_act_ overall poorly correlated with Δ*p*_conv_  (*r* = 0.35). However, color M-mode-derived gradients (which included both convective and inertance components) correlated closely with actual pressure gradients (*y* = 0.95*x* + 0.24, *r* = 0.96) [11]. These findings are consistent with previous canine, human, and numerical modeling studies in which, under normal loading conditions, ignoring the inertance components underestimated transmitral gradients by as much as 12 mmHg [9, 12, 13]. 

This data suggests that in “sicker” patients (i.e., those with greater left atrial volumes, mitral valve dysfunction, and abnormal filling pressures) the inertance contribution to transmitral pressure gradients is greater and thereby implying the Doppler-derived gradients significantly underestimate, and more so with larger LA volume, the actual gradient. Conversely, a decreased pulmonary venous *S*/*D* ratio in the setting of “normal” transmitral Doppler waveforms, which is a marker for heart failure [15], predicts a lower inertial component to the actual transmitral gradient, and hence the convective term more closely approximates the true gradient. These findings and concepts are consistent with separate studies performed by Nakatani et al. [12] in which physiologic predictors of transmitral *M* included systolic *LV* pressures and actual transmitral gradients. 

Flachskampf and colleagues, in an *in vitro* model, showed that inertance depended on the orifice diameter and conduit length more than the actual gradients. This explains, in part, the basis of the limitations of the modified Bernoulli equation in larger orifices and lower pressure/velocity scenarios, like a normal mitral valve. In the context of the heart, he suggested that *M* was a function of geometrical characteristics of the mitral valve area and apparatus length [10]. Even though these results might appear contradictory, chronic adverse changes in ventricular loading conditions are linked to pathologic changes in the mitral valve function and geometry. 

While these experiments demonstrate the role of nonconvective, or inertial, forces for transmitral flow, it is important to consider that the same principles apply for other applications that measure pressure gradients within the heart, such as intraventricular pressure gradients [16, 17], right ventricular filling pressures [18], intracardiac shunts, and pulmonary hypertension [19]. For example, while Doppler velocities are routinely used to derive pulmonary artery pressures [20], it is well known that these pressures typically correlate poorly with actual catheter-derived measurements [21]. In these studies, even when right atrial pressures are included in these estimates, there is still a significant (~8 mmHg) source of error in more than 50% of patients that cannot be explained by routinely measured clinical parameters and are hypothesized to be only accounted for by nonconvective forces [22]. Clearly, further studies are needed to substantiate and better understand these complex complementary or conflict determinants to intracardiac pressure gradients. 

## 6. Conclusions

The use of echocardiography in the evaluation of cardiac disease and the critically ill is becoming ubiquitous. It is standard of care for intraoperative management of patients undergoing valve surgery without contraindications [23]. Unfortunately, clinically useful tools to accurately quantitatively determine the contributing factors to nonconvective forces, or the inertial components of intracardiac blood flow, are lacking which potentially further explains why this parameter is typically ignored when determining intracardiac pressure gradients. Nevertheless, as outlined, these nonconvective forces remain a variable and critical contribution to the determination of pressure gradients. A thorough understanding of the principles of fluid dynamics, the limitations of Doppler echocardiography, and the assumptions of the modified Bernoulli equation are critical in accurate interpretation of clinical data.
